# Supplementary material for: The Systems Biology Research Tool: evolvable open-source software
Source: BMC Syst Biol. 2008 Jun 29;2:55. doi: 10.1186/1752-0509-2-55 (PMC2446383; doi:10.1186/1752-0509-2-55)
Supplement: Additional file 1 — SBRT Archive. An archive of the current version of the Systems Biology Research Tool. [file 1752-0509-2-55-S1.zip › sbrt-1.4.0/doc/developers_guide/api/sbrt/kernel/fba/analysis/FluxIntervalAnalyzer.html]

FluxIntervalAnalyzer


|  |  |  |
| --- | --- | --- |
| **Overview** \| **Package** \| **Class** \| **Use** \| **Tree** \| **Deprecated** \| **Index** \| **Help** | |  |
| PREV CLASS   NEXT CLASS | **FRAMES**    **NO FRAMES**     **All Classes** |  |
| SUMMARY: NESTED \| FIELD \| CONSTR \| METHOD | DETAIL: FIELD \| CONSTR \| METHOD |  |


---


## sbrt.kernel.fba.analysis Class FluxIntervalAnalyzer

```
java.lang.Object
  sbrt.kernel.fba.analysis.FluxIntervalAnalyzer
```

---

``` public class FluxIntervalAnalyzer extends java.lang.Object ```

This class is used to analyze flux intervals.

The `zeroCutoff` arguments to each method must be:

- Greater than zero.
- Not `Double.NaN`.
- Not infinite.

**Author:**
:   This class was written and documented by Jeremiah Wright while in the
    Wagner lab.

---

**Constructor Summary**

`FluxIntervalAnalyzer()`
  
          Constructs a new flux interval analyzer.


| **Method Summary** | |
| --- | --- |
| `java.util.Set<java.lang.String>` | `getAlwaysActive(java.util.Map<java.lang.String,Interval> fluxIntervals, double zeroCutoff)`             Returns the always-active fluxes in the provided map. |
| `java.util.Set<java.lang.String>` | `getNeverActive(java.util.Map<java.lang.String,Interval> fluxIntervals, double zeroCutoff)`             Returns the never-active fluxes in the provided map. |
| `java.util.Set<java.lang.String>` | `getPotentiallyActive(java.util.Map<java.lang.String,Interval> fluxIntervals, double zeroCutoff)`             Returns the potentially-active fluxes in the provided map. |
| `int` | `lowerBoundsEqualZero(java.util.Collection<Interval> intervals, double zeroCutoff)`             Returns the number of provided intervals whose lower bound equals zero. |
| `int` | `lowerBoundsEqualZero(java.util.Map<?,Interval> intervals, double zeroCutoff)`             Returns the number of provided intervals whose lower bound equals zero. |
| `int` | `lowerBoundsGreaterThanZero(java.util.Collection<Interval> intervals, double zeroCutoff)`             Returns the number of provided intervals whose lower bounds are greater than zero. |
| `int` | `lowerBoundsGreaterThanZero(java.util.Map<?,Interval> intervals, double zeroCutoff)`             Returns the number of provided intervals whose lower bounds are greater than zero. |
| `int` | `rangesEqualZero(java.util.Collection<Interval> intervals, double zeroCutoff)`             Returns the number of provided intervals whose ranges equal zero. |
| `int` | `rangesEqualZero(java.util.Map<?,Interval> intervals, double zeroCutoff)`             Returns the number of provided intervals whose ranges equal zero. |
| `int` | `rangesGreaterThanZero(java.util.Collection<Interval> intervals, double zeroCutoff)`             Returns the number of provided intervals whose ranges are greater than zero. |
| `int` | `rangesGreaterThanZero(java.util.Map<?,Interval> intervals, double zeroCutoff)`             Returns the number of provided intervals whose ranges are greater than zero. |
| `int` | `upperBoundsEqualZero(java.util.Collection<Interval> intervals, double zeroCutoff)`             Returns the number of provided intervals whose upper bounds equal zero. |
| `int` | `upperBoundsEqualZero(java.util.Map<?,Interval> intervals, double zeroCutoff)`             Returns the number of provided intervals whose upper bounds equal zero. |
| `int` | `upperBoundsGreaterThanZero(java.util.Collection<Interval> intervals, double zeroCutoff)`             Returns the number of provided intervals whose upper bounds are greater than zero. |
| `int` | `upperBoundsGreaterThanZero(java.util.Map<?,Interval> intervals, double zeroCutoff)`             Returns the number of provided intervals whose upper bounds are greater than zero. |

**Methods inherited from class java.lang.Object**

`clone, equals, finalize, getClass, hashCode, notify, notifyAll, toString, wait, wait, wait`

**Constructor Detail**

### FluxIntervalAnalyzer

```
public FluxIntervalAnalyzer()
```

:   Constructs a new flux interval analyzer.


**Method Detail**

### getAlwaysActive

```
public java.util.Set<java.lang.String> getAlwaysActive(java.util.Map<java.lang.String,Interval> fluxIntervals,
                                                       double zeroCutoff)
```

:   Returns the always-active fluxes in the provided map.

    :   **Parameters:**: `fluxIntervals` - reaction names mapped to their corresponding flux interval.: `zeroCutoff` - the amount by which a value can differ from zero, but still be considered equal to zero. **Returns:**: the keys in the provided map whose corresponding interval has a lower bound greater than zero.

---


### getPotentiallyActive

```
public java.util.Set<java.lang.String> getPotentiallyActive(java.util.Map<java.lang.String,Interval> fluxIntervals,
                                                            double zeroCutoff)
```

:   Returns the potentially-active fluxes in the provided map.

    :   **Parameters:**: `fluxIntervals` - reaction names mapped to their corresponding flux interval.: `zeroCutoff` - the amount by which a value can differ from zero, but still be considered equal to zero. **Returns:**: the keys in the provided map whose corresponding interval has a lower bound equal to zero and an upper bound greater than zero.

---


### getNeverActive

```
public java.util.Set<java.lang.String> getNeverActive(java.util.Map<java.lang.String,Interval> fluxIntervals,
                                                      double zeroCutoff)
```

:   Returns the never-active fluxes in the provided map.

    :   **Parameters:**: `fluxIntervals` - reaction names mapped to their corresponding flux interval.: `zeroCutoff` - the amount by which a value can differ from zero, but still be considered equal to zero. **Returns:**: the keys in the provided map whose corresponding interval has lower and upper bounds equal to zero.

---


### lowerBoundsEqualZero

```
public int lowerBoundsEqualZero(java.util.Map<?,Interval> intervals,
                                double zeroCutoff)
```

:   Returns the number of provided intervals whose lower bound equals zero.

    :   **Parameters:**: `intervals` - a map whose values are intervals.: `zeroCutoff` - the amount by which a value can differ from zero, but still be considered equal to zero. **Returns:**: the number of provided intervals whose lower bound equals zero.

---


### lowerBoundsEqualZero

```
public int lowerBoundsEqualZero(java.util.Collection<Interval> intervals,
                                double zeroCutoff)
```

:   Returns the number of provided intervals whose lower bound equals zero.

    :   **Parameters:**: `intervals` - a collection of intervals.: `zeroCutoff` - the amount by which a value can differ from zero, but still be considered equal to zero. **Returns:**: the number of provided intervals whose lower bound equals zero.

---


### lowerBoundsGreaterThanZero

```
public int lowerBoundsGreaterThanZero(java.util.Map<?,Interval> intervals,
                                      double zeroCutoff)
```

:   Returns the number of provided intervals whose lower bounds are greater
    than zero.

    :   **Parameters:**: `intervals` - a map whose values are intervals.: `zeroCutoff` - the amount by which a value can differ from zero, but still be considered equal to zero. **Returns:**: the number of provided intervals whose lower bounds are greater than zero.

---


### lowerBoundsGreaterThanZero

```
public int lowerBoundsGreaterThanZero(java.util.Collection<Interval> intervals,
                                      double zeroCutoff)
```

:   Returns the number of provided intervals whose lower bounds are greater
    than zero.

    :   **Parameters:**: `intervals` - a collection of intervals.: `zeroCutoff` - the amount by which a value can differ from zero, but still be considered equal to zero. **Returns:**: the number of provided intervals whose lower bounds are greater than zero.

---


### upperBoundsEqualZero

```
public int upperBoundsEqualZero(java.util.Map<?,Interval> intervals,
                                double zeroCutoff)
```

:   Returns the number of provided intervals whose upper bounds equal zero.

    :   **Parameters:**: `intervals` - a map whose values are intervals.: `zeroCutoff` - the amount by which a value can differ from zero, but still be considered equal to zero. **Returns:**: the number of provided intervals whose upper bounds equal zero.

---


### upperBoundsEqualZero

```
public int upperBoundsEqualZero(java.util.Collection<Interval> intervals,
                                double zeroCutoff)
```

:   Returns the number of provided intervals whose upper bounds equal zero.

    :   **Parameters:**: `intervals` - a collection of intervals.: `zeroCutoff` - the amount by which a value can differ from zero, but still be considered equal to zero. **Returns:**: the number of provided intervals whose upper bounds equal zero.

---


### upperBoundsGreaterThanZero

```
public int upperBoundsGreaterThanZero(java.util.Map<?,Interval> intervals,
                                      double zeroCutoff)
```

:   Returns the number of provided intervals whose upper bounds are greater
    than zero.

    :   **Parameters:**: `intervals` - a map whose values are intervals.: `zeroCutoff` - the amount by which a value can differ from zero, but still be considered equal to zero. **Returns:**: the number of provided intervals whose upper bounds are greater than zero.

---


### upperBoundsGreaterThanZero

```
public int upperBoundsGreaterThanZero(java.util.Collection<Interval> intervals,
                                      double zeroCutoff)
```

:   Returns the number of provided intervals whose upper bounds are greater than zero.

    :   **Parameters:**: `intervals` - a collection of intervals.: `zeroCutoff` - the amount by which a value can differ from zero, but still be considered equal to zero. **Returns:**: the number of provided intervals whose upper bounds are greater than zero.

---


### rangesEqualZero

```
public int rangesEqualZero(java.util.Map<?,Interval> intervals,
                           double zeroCutoff)
```

:   Returns the number of provided intervals whose ranges equal zero.

    :   **Parameters:**: `intervals` - a map whose values are intervals.: `zeroCutoff` - the amount by which a value can differ from zero, but still be considered equal to zero. **Returns:**: the number of provided intervals whose ranges equal zero.

---


### rangesEqualZero

```
public int rangesEqualZero(java.util.Collection<Interval> intervals,
                           double zeroCutoff)
```

:   Returns the number of provided intervals whose ranges equal zero.

    :   **Parameters:**: `intervals` - a collection of intervals.: `zeroCutoff` - the amount by which a value can differ from zero, but still be considered equal to zero. **Returns:**: the number of provided intervals whose ranges equal zero.

---


### rangesGreaterThanZero

```
public int rangesGreaterThanZero(java.util.Map<?,Interval> intervals,
                                 double zeroCutoff)
```

:   Returns the number of provided intervals whose ranges are greater than zero.

    :   **Parameters:**: `intervals` - a map whose values are intervals.: `zeroCutoff` - the amount by which a value can differ from zero, but still be considered equal to zero. **Returns:**: the number of provided intervals whose ranges are greater than zero.

---


### rangesGreaterThanZero

```
public int rangesGreaterThanZero(java.util.Collection<Interval> intervals,
                                 double zeroCutoff)
```

:   Returns the number of provided intervals whose ranges are greater than zero.

    :   **Parameters:**: `intervals` - a collection of intervals.: `zeroCutoff` - the amount by which a value can differ from zero, but still be considered equal to zero. **Returns:**: the number of provided intervals whose ranges are greater than zero.


---


|  |  |  |
| --- | --- | --- |
| **Overview** \| **Package** \| **Class** \| **Use** \| **Tree** \| **Deprecated** \| **Index** \| **Help** | |  |
| PREV CLASS   NEXT CLASS | **FRAMES**    **NO FRAMES**     **All Classes** |  |
| SUMMARY: NESTED \| FIELD \| CONSTR \| METHOD | DETAIL: FIELD \| CONSTR \| METHOD |  |


---
